# How to undertake procedures while on antiplatelet agents: a hematologist's view

**DOI:** 10.1016/j.rpth.2024.102539

**Published:** 2024-08-05

**Authors:** Dawn Swan, Robert Turner, James Douketis, Jecko Thachil

**Affiliations:** 1 Department of Haematology, Austin Health, Melbourne, Victoria, Australia; 2 Department of Intensive Care, St Vincent’s Hospital, Melbourne, Victoria, Australia; 3 Department of Medicine, St. Joseph’s Healthcare Hamilton and McMaster University, Hamilton, Ontario, Canada; 4 Manchester Academic Health Science Centre, University of Manchester, Manchester, United Kingdom

**Keywords:** anesthesia, antiplatelet, aspirin, bleeding, clopidogrel, prasugrel, surgery, ticagrelor

## Abstract

Cardiovascular diseases (CVDs) are the leading cause of mortality globally while also contributing to excess health system costs. Significant advancements have been made in the understanding and prevention of deaths from CVD. In addition to risk factor modifications, one of the key developments in this area is the appropriate prescribing of antiplatelet medications for secondary prevention of CVD. With the advent of vascular devices, there has been an increased use of potent antiplatelet agents to mitigate thrombosis risk. A well-recognized, albeit rare complication of antiplatelet drugs is the heightened risk of bleeding. This adverse effect is particularly relevant when a patient receiving these medications may require an urgent surgery. In addition, for elective surgeries, although these drugs can be withheld, there may be some situations when interruption of antiplatelet agents, even for short duration, may lead to thrombotic events. There are no robust guidelines on how to manage these clinical scenarios, although there have been some important studies published recently in this area. In this review, we provide our approach to patients on antiplatelet drugs who may require urgent surgeries or surgical interventions.

## Essentials

• Several guidelines provide recommendations concerning interruption of antiplatelet therapy for elective procedures or interventions.
• This articles aims to provide an update on these recommendations as well as including pragmatic approaches for urgent cases.
• Bridging therapy therapy may be required for select situations where there is high thrombotic risk. The evidence base underpinning this is not rigorous, and requires case-by-case multidisciplinary discussion.
• Aiming to minimise interruption of antiplatelet therapy is recommended for the majority of lower thrombotic risk cases.

## Graphical abstract

**Figure:**
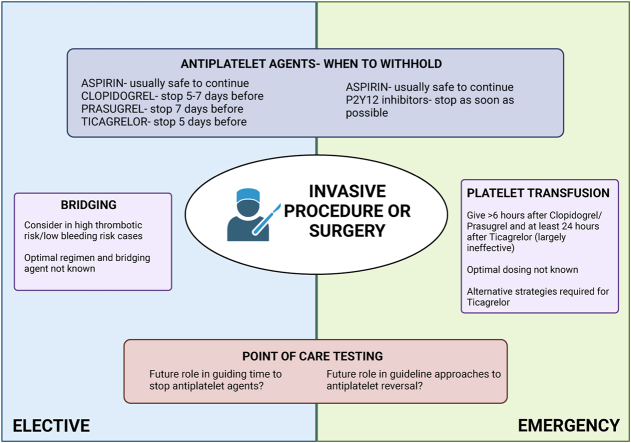

## 1 Introduction

The global burden of diseases, injuries, and risk factors study, which has tracked trends in death and disability since 1990, reports that cardiovascular disease (CVD) remains the leading cause of morbidity and mortality worldwide [1]. The global prevalence of CVD nearly doubled from 271 million people in 1990 to 523 million in 2019, with a proportionally smaller increase in mortality, from 12.1 to 18.6 million [2]. Since the advent of routine percutaneous coronary intervention (PCI) and a worldwide increased focus on cardiovascular risk factor modification (hypertension, hyperlipidemia, diabetes mellitus, smoking cessation, etc.), deaths from CVD have actually decreased by over 50% in some countries [[3], [4], [5], [6]]. Alongside these measures, antiplatelet therapy forms a therapeutic cornerstone for the management of CVD, and, as such, prescriptions for P2Y_12_ inhibitors have increased significantly in recent years [7]. The issue of management of patients who are receiving antiplatelet therapy and require a surgery or procedure is common. In a higher-risk group of patients with recent PCI or coronary stenting, 4% to 8% of patients will require major surgery in the year that follows [8].

Against this background, the aim of this review was to assess the available evidence and published guidelines that inform the management of patients who are taking antiplatelet therapy and require an elective or urgent surgery. Specifically, we also consider evidence relating to the role of antiplatelet bridging agents, platelet transfusions, and antiplatelet reversal agents in this clinical setting [9].

## 2 The Antiplatelet Agents and their Mechanism of Action

The most frequently prescribed antiplatelet drugs are aspirin and the P2Y_12_ inhibitors clopidogrel, ticagrelor, and, to a lesser extent, prasugrel, which is usually only chosen in certain high-risk patient groups [10]. The pharmacologic properties of these and other antiplatelet agents are shown in Table 1 [[11], [12], [13]]. Antiplatelet monotherapy usually comprises aspirin or clopidogrel, whereas dual antiplatelet therapy (DAPT) refers to the combination of aspirin with a P2Y_12_ inhibitor. Briefly, aspirin derives antiplatelet activity by irreversible acetylation of serine-530 of the COX-1 enzyme, leading to near-complete inhibition of thromboxane A2 production, preventing prostanoid-mediated platelet aggregation [14]. The P2Y_12_ inhibitors prevent adenosine 5' diphosphate (ADP) from binding to the P2Y_12_ receptor, impeding subsequent exposure of glycoprotein (GP) IIb/IIIa and thereby preventing fibrinogen-mediated platelet activation [15].

**Table 1:** Pharmacokinetic properties of the antiplatelet agents available and in clinical trials.

Drug	Mechanism of action	Route of administration	Reversible or irreversible	Half-life	Time to recover platelet function after drug withdrawal
**Aspirin**	COX-1 inhibitor	Oral	Irreversible	15-20 min	30% at 48 h
**Clopidogrel**	P2Y_12_ inhibitor	Oral	Irreversible	6-8 h	40% at 3 d
**Prasugrel**			Irreversible	7 h	2-3 d
**Ticagrelor**			Reversible	7-9 h	57% at 24 h
**Ticlopidine**			Irreversible	8-12 h	3-14 d
**Abciximab**	GPIIb/IIIa inhibitor	i.v.	Reversible	10-15 min	12 h
**Eptifibatide**				2.5 h	2-4 h
**Tirofiban**				2 h	2-4 h
**Cangrelor**				3-6 min	3-6 min
**Vorapaxar**	Protease-activated receptor-1 inhibitors	Oral	Reversible	5-13 d	4-8 wk
**Cilostazol**	Phosphodiesterase inhibitors-3 or -5 inhibitors	Oral	Reversible	11 h	12-16 h
**Dipyridamole**		Sildenafil and dipyridamole can be i.v.		10-12 h	-
**Sildenafil**				4-6 h	-
**Tadalafil**				17.5 h	24-36 h
**Vardenafil**				5 h	-
**Iloprost**	PGI_2_ analog	i.v.	Reversible	20-30 min	2 h
**Epoprostenol**			Reversible	<1 min	<10 min
**BMS-986120 and BMS-986141**	Protease-activated receptor-4 antagonist	In trials	Reversible	45-84 h 34-45 h	∼24 h >24 h
**Anfibatide**	GPIb-V-IX complex inhibitor		Reversible	5-7 h	8 h
**Revacept(advanceCOR)**	GPVI competitive inhibitor		Reversible	87-137 h	48 h
**Glenzocimab**	GPVI inhibitor-Ab		Reversible	9.6 h	25%-55% 24 h
**VLX-1005**	12-Lipoxygenase inhibitor		Irreversible	2.9 h	-

Ab, antibody; GP, glycoprotein; i.v., intravenous.

## 3 Elective Surgery and Low-Risk Interventional Radiology Procedures

The perioperative management of patients taking antiplatelet agents involves multiple considerations, including the indication for antiplatelet use (eg, primary vs secondary prevention), use of DAPT or monotherapy, the urgency of surgery, and associated bleeding and thrombotic risks [16,17]. Risk prediction tools such as the Revised Cardiac Risk Index and the National Surgical Quality Improvement Program perform poorly at predicting major adverse cardiac events (MACEs) and are not validated for predicting MACEs in the context of perioperative withholding of antiplatelet agents. Similarly, the CHA_2_DS_2_-VASc score has poor predictive utility for perioperative stroke [[18], [19], [20], [21], [22]]. The American Heart Association and European Society of Cardiology (ESC)/European Society of Anaesthesiology and Intensive Care guidelines for perioperative evaluation of cardiac risk recommend utilizing a Bayesian inference approach that incorporates patient and surgical factors [23,24]. Similarly, predicting surgical-associated bleeding risk on a broad level is challenging as scores such as HAS-BLED and the International Society on Thrombosis and Haemostasis Bleeding Assessment Tool have poor utility in this clinical setting [21]. Limited evidence suggests that specific surgery types (eg, cancer surgery) and selected comorbidities (eg, prior bleeding) may affect perioperative bleeding risk [25,26]. Empiric classifications of surgery and procedure types according to bleeding risk are available to estimate individual bleeding [27]. Patient-related factors that may affect bleeding risk include concomitant renal or liver disease, use of nonsteroidal anti-inflammatory drugs, and other medications that impair hemostasis [9].

### 3.1 Aspirin

There is uncertainty as to the optimal perioperative management of patients taking antiplatelet monotherapy with aspirin (ASA) in patients having a low-to-moderate bleeding risk surgery, whereas interruption is suggested for interventions associated with very high bleeding risk, such as intracranial and spinal surgeries or neuraxial/deep nerve root procedures [21,[28], [29], [30], [31], [32], [33]]. The POISE-2 trial, which involved 10,010 patients having elective noncardiac surgery (most commonly orthopedic, general, or urological/gynecologic procedures), randomized allocated patients to continue or stop ASA (continuation stratum) or to initiate or not take ASA (initiation stratum) perioperatively [34]. Although the group that took ASA perioperatively experienced a significantly higher incidence of major bleeding (4.6% vs 3.8%; hazard ratio, 1.2; 95% CI, 1.01-1.50), there was no significant difference in bleeding among patients in the continuation stream (4.6% vs 4.1%; hazard ratio, 1.1; 95% CI, 0.84-1.48), who either continued or stopped ASA, and this subgroup may be most representative of patients assessed in practice who are already taking ASA. In a subgroup of 470 patients who had undergone PCI and stenting, perioperative ASA continuation was associated with significantly lower rate of nonfatal myocardial infarction (MI) at 30 days (absolute risk reduction, 5.9%; 95% CI, 1.0%-10.8%) without a significant increase in major and life-threatening bleeding [35]. If a clinical decision is made to withhold aspirin prior to high-risk bleeding procedures, adequate recovery of platelet function for hemostasis can be expected within 3 to 5 days following cessation [[36], [37], [38]]. The ESC and 2022 American College of Chest Physician (CHEST) guidelines suggest consideration of ASA continuation according to individual bleeding and thrombosis risk; if ASA interruption occurs, it is recommended to stop ≤7 days prior to high bleeding risk surgery, whereas the British Society for Haematology guidelines and the French Working Group on Perioperative Haemostasis in collaboration with the French Society of Anaesthesia and Intensive Care Medicine suggest 3 days of ASA interruption is sufficient [[28], [29], [30],^39^]. For patients taking high-dose ASA, the American Society of Regional Anesthesia and Pain Medicine, European Society of Regional Anaesthesia and Pain Therapy, and Society of Interventional Radiology recommend discontinuing aspirin ≥3 days prior to a planned intervention [31,33].

### 3.2 P2Y_12_ inhibitors

DAPT is commonly used in patients who have undergone PCI with coronary stent insertion. Up to 14% of these patients may require surgery in the year following PCI [40]. Rates of MACEs (death, MI, and stent thrombosis) are particularly high during the first 6 weeks after stenting, at 10% to 45%, but fall to 4% at 6 months and return to preprocedural levels after 12 months [41]. Such risks are dependent upon preexisting comorbidities and surgical factors [20], as well as stent-related factors [42,43].

Timing of discontinuation depends upon drug pharmacokinetics and patient and procedural bleeding risk. In the CURE study of ASA and clopidogrel compared with ASA monotherapy in unstable angina, 2072 individuals underwent coronary artery bypass grafting (CABG). If clopidogrel was discontinued ≥5 days preoperatively along with ASA continuation, this was not associated with an increased risk of bleeding observed compared with continuing ASA alone [44]. In 1261 patients with acute coronary syndrome (ACS) requiring cardiac surgery, DAPT with aspirin and ticagrelor was associated with similar rates of bleeding in the PLATO study compared with aspirin/clopidogrel. In this study, ticagrelor was held 1 to 3 days before surgery and clopidogrel was held 5 days before surgery. Aspirin was continued throughout [45]. In comparison, the addition of prasugrel was associated with significantly more bleeding, as determined by 12-hour chest tube loss in 346 patients undergoing CABG within the TRITON-TIMI trial [46]. In addition, platelet inhibition persists for longer following prasugrel cessation [47].

The majority of the current major guidelines recommend that ticagrelor and clopidogrel should be ceased 5 days before surgery, and prasugrel requires 7 days, albeit with very low quality of evidence [28,29,39]. The 2022 CHEST guideline suggests 3 to 5 days for ticagrelor, whereas the current iteration of the ESC guideline has reverted to 5 days, having previously suggested that 3 days is sufficient [30,43].

For patients who require noncardiac surgery with a history of recent MI or who have undergone PCI and stenting, surgery should ideally be postponed until DAPT is no longer required [29]. Both the ESC and American Heart Association currently recommend 6 months of DAPT after PCI and 1 year following ACS [48]. While the need for DAPT immediately post-PCI was reaffirmed by the recent STOPDAPT-3 study, which demonstrated prasugrel monotherapy to be associated with increased rates of in-stent thrombosis compared with DAPT [49], a number of other trials have demonstrated that 1 to 3 months of DAPT is likely sufficient. A meta-analysis of 4 such studies (MASTER-DAPT, TWILIGHT, TICO, and STOPDAPT-2 [[50], [51], [52], [53]]) reported no increase in MACEs or in-stent thrombosis, with reduced levels of major and clinically relevant nonmajor bleeding compared with standard duration DAPT [54].

Current recommendations suggest that a minimum period of 1 month should have elapsed after elective PCI and 3 months after ACS before a patient has nonemergency noncardiac surgery [29]. Should future guidelines shorten the required period of dual antiplatelets, temporary discontinuation of P2Y_12_ inhibition may be required for significantly fewer patients. If surgery is required within 1 month of PCI and stenting, bridging may be a consideration, as discussed later. A summary of the guideline recommendations for elective procedures is shown in Table 2.

**Table 2:** Guideline recommendations in elective surgery.

Guideline	Elective procedures	Neuraxial procedures	Bridging
ESC/European Society of Anaesthesiology and Intensive Care 2022	Continue aspirin Clopidogrel: discontinue 5 d prior Ticagrelor: 3-5 d prior Prasugrel: 7 d prior	No specific recommendations	Consider in high-risk cases with cangrelor, tirofiban, or eptifibatide
ESC/European Association for Cardio-Thoracic Surgery 2017	Continue aspirin Clopidogrel: discontinue 5 d prior Ticagrelor: 3 d prior Prasugrel: 7 d prior	No specific recommendations	Consider if surgery is <1 mo after stent insertion
ESC/European Society of Anaesthesiology 2014	Continue aspirin	No specific recommendations	Consider for very high thrombotic risk patients
	Clopidogrel: discontinue 5 d prior		
	Ticagrelor: 3-5 d prior		
	Prasugrel: 7 d prior		
British Society for Haematology 2016	Continue aspirin Clopidogrel: discontinue 5 d prior Ticagrelor: 5 d prior Prasugrel: 7 d prior	Continue aspirin Discontinue P2Y_12_ inhibitors 7 d prior	No specific recommendations
American College of Chest Physicians 2022	Continue aspirin Clopidogrel: discontinue 5 d prior Ticagrelor: 3-5 d prior Prasugrel: 7 d prior	No specific recommendations	Not routinely recommended; consider in select high-risk procedures
Japanese Circulation Society 2020	Continue aspirin Clopidogrel: discontinue 5 d prior Ticagrelor: 3 d prior Prasugrel: 7 d prior	No specific recommendations	Not recommended
Clinical Excellence Commission, Australia 2018	Continue aspirin Clopidogrel: discontinue 7 d prior Ticagrelor: 5 d prior Prasugrel: 7 d prior Ticlopidine: 14 d prior	Continue aspirin Clopidogrel: discontinue 5 d prior Ticagrelor: 5 d prior Prasugrel: 7 d prior Ticlopidine: 14 d prior	Not recommended

ESC, European Society of Cardiology.

## 4 Neuraxial Anesthesia/Deep Plexus Regional Anesthesia/High-Risk interventional Radiology/Pain Procedures

**Figure 1:**
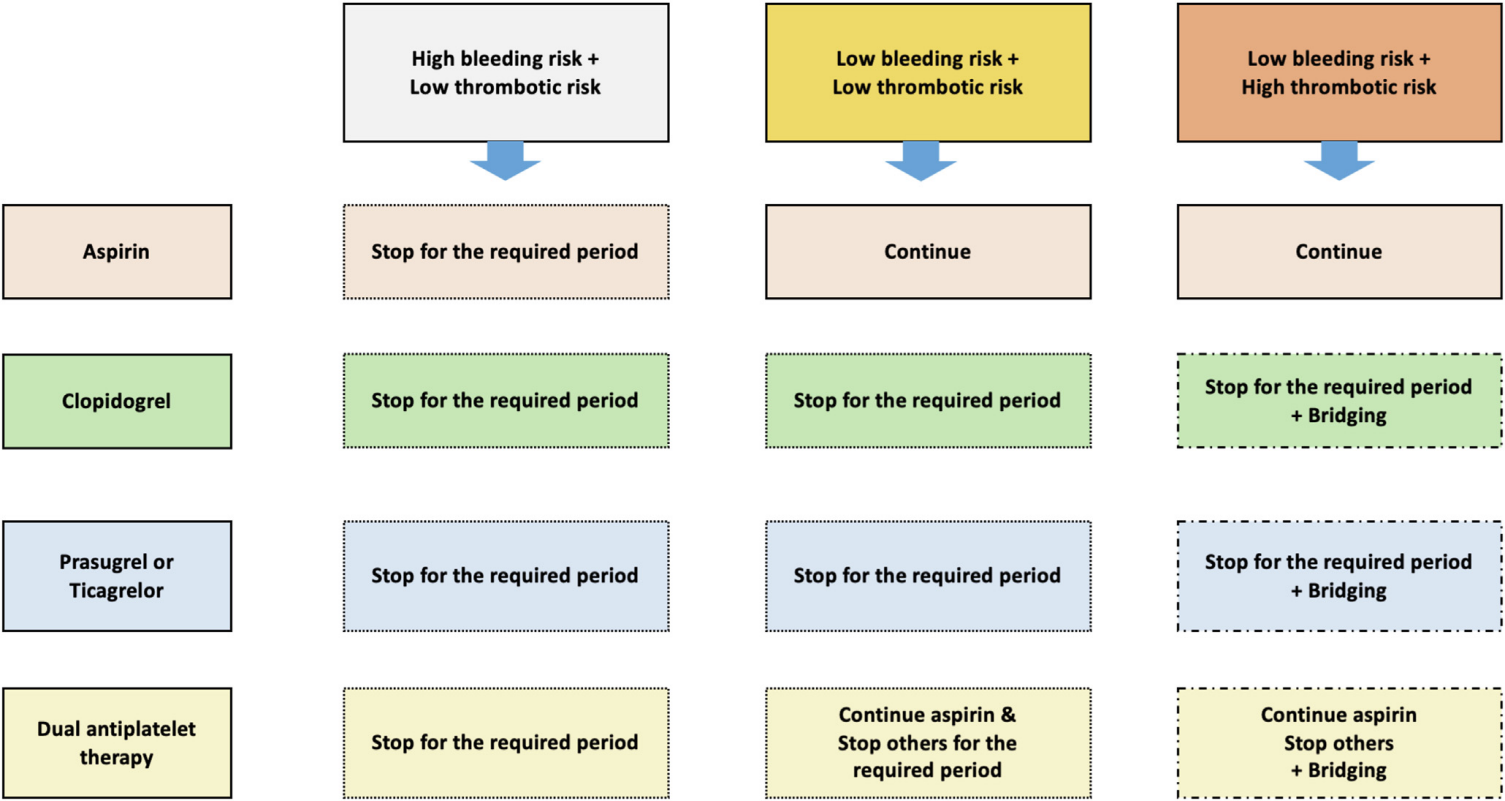
An algorithm for the management of antiplatelet agents around surgery or interventions.

The main concern as to the safety of using antiplatelet agents around neuraxial procedures relates to the risk for spinal hematoma, a rare but potentially devastating complication [55]. However, relevant data are limited. There was no increase in expected bleeding following spinal or epidural anesthesia in 193 patients receiving low-dose ASA prior to orthopedic surgery [56], nor was bleeding increased after epidural anesthesia with ASA use for preeclampsia in 1422 women compared with 1361 who were allocated placebo. In this study, patients continued antiplatelet therapy until the day of delivery [57]. There is less available evidence regarding the P2Y_12_ inhibitors. Guidelines either do not address this question or recommend a similar discontinuation as that for elective surgery [28,29,33,39,58]. The evidence for deep plexus blockade, high-risk interventional radiology, and pain procedures is also scarce, and guidelines recommend following guidance for neuraxial blockade due to lack of evidence in this field [21,[31], [32], [33]]. A general approach to patients on DAPT who require elective procedures or neuraxial anesthesia is shown in Table 3 and Figure 1. A summary of the guideline recommendations is shown in Table 4.

**Table 3:** Author’s recommendations for elective surgery and neuraxial anesthesia.

Drug	Elective surgery	Neuraxial anesthesia
Aspirin	Continue unless very high risk of bleeding, then stop 5 d before	No contraindication following a risk-benefit analysis
Clopidogrel	5-7 d	5-7 d
Prasugrel	7 d	7 d
Ticagrelor	5 d	5 d

**Table 4:** Guideline recommendations in neuraxial anesthesia/deep plexus regional anesthesia/high-risk interventional radiology and pain procedures.

Guideline	Neuraxial anesthesia/deep plexus regional anesthesia/high-risk interventional radiology/pain procedures
The Canadian Association for Interventional Radiology and The Cardiovascular and Interventional Radiological Society of Europe 2019	Continue aspirin
	Discontinue P2Y_12_ inhibitors 5 d prior
ESAIC/ESRA 2022	Continue low-dose aspirin High-dose aspirin: discontinue 3-7 d prior Clopidogrel: 7 d prior Ticagrelor: 5-7 d prior Prasugrel: 7 d prior
American Society of Regional Anesthesia and Pain Medicine, European Society of Regional Anaesthesia and Pain Medicine, American Academy of Pain Medicine, International Neuromodulation Society, North American Neuromodulation Society, and World Institute of Pain 2018	Aspirin: discontinue 6 d prior to high bleeding risk procedures Clopidogrel: discontinue 7 d prior to moderate-high bleeding risk procedures (5 d in high thrombotic risk cases) Ticagrelor: 5 d prior to moderate-high bleeding risk procedures Prasugrel: 7-10 d prior to moderate-high bleeding risk procedures
American Society of Regional Anesthesia and Pain Medicine 2018	Continue aspirin Clopidogrel: discontinue 7 d prior Ticagrelor: 5 d prior Prasugrel: 7-10 d prior Ticlopidine: 10 d prior
European Society of Anaesthesiology 2010	Continue aspirin Clopidogrel: discontinue 7 d prior Ticagrelor: 5 d prior Prasugrel: 7-10 d prior Ticlopidine: 10 d prior

ESAIC, European Society of Anaesthesiology and Intensive Care; ESRA, European Society of Regional Anaesthesia & Pain Therapy; IR, interventional radiology.

## 5 Bridging

Bridging in the context of perioperative DAPT management refers to interrupting the P2Y_12_ inhibitor ± ASA and starting an intravenous short-acting antiplatelet agent, which is stopped shortly before surgery. Bridging agents include the GPIIb/IIIa receptor antagonists eptifibatide and tirofiban and the intravenous P2Y_12_ inhibitor cangrelor. The evidence in support of this strategy was recently reviewed extensively [59], with most data derived from uncontrolled observational studies.

### 5.1 Eptifibatide

In a propensity-matched analysis of 68 patients who had CABG surgery after PCI and were bridged with eptifibatide, there was no difference reported in rates of red cell or platelet transfusion requirements, but tamponade requiring reoperation was significantly more frequent in the bridged than in the nonbridged group (10% vs 3%). MACE was reported at 7% in the bridged patients and was not published for the control group [60]. Another small study compared 30 patients considered high-risk for cardiovascular events who were bridged with eptifibatide before thoracic surgery with 69 matched controls who did not receive bridging. There was no difference observed in rates of blood product requirements, bleeding, MI, or cardiovascular death. However, the bridged cohort was significantly enriched for the presence of stents, stents placed within the previous year, and clopidogrel administration, suggesting a higher thrombotic risk group [61].

### 5.2 Tirofiban

Only 1 study using tirofiban for bridging was a retrospective cohort study of 87 patients with prior PCI and stenting within the previous 12 months who were bridged with tirofiban and who were compared with 227 control patients. There was no significant difference in death, MI, or transfusion requirements between the 2 groups. There was a nonsignificant reduction in MACEs between the bridged and nonbridged groups (2.3% vs 7.5%; *P* = .08) and a significant reduction of net adverse clinical events comprising a composite of MACEs and bleeding with bridging (8.0% vs 22.5%; *P* < .01), but this later benefit was limited to patients who had stenting within 60 days of surgery [62].

### 5.3 Cangrelor

Cangrelor, the only available intravenous P2Y_12_ inhibitor, has advantages over the GPIIb/IIIa inhibitors, including no dose adjustment required in renal impairment [63] and availability of a specific bridging dose and protocol, whereas eptifibatide and tirofiban rely on dosing used in patients with an ACS. The BRIDGE trial randomized 210 patients with ACS or a coronary stent, who required elective CABG, to cangrelor bridging or placebo with a primary safety endpoint of bleeding. There was no significant difference in CABG-related bleeding identified, and rates of thrombotic complications were low, occurring in 2.8% and 4% of the cangrelor and placebo arms respectively, although the study was not powered to assess thrombotic outcomes. Additionally, only half the patients had been stented previously, and the timeframe between PCI and surgery was not reported [64].

In highly selected patients with a high thrombotic risk and a low bleeding risk, bridging with a short-acting antiplatelet agent may be considered. Recommendations from practice guidelines vary widely. The ESC/European Association for Cardio-Thoracic Surgery and ESC/European Society of Anaesthesiology guidelines from 2017 and 2014 [43,65], respectively, suggest consideration of bridging with an intravenous antiplatelet agent if DAPT is discontinued in high-risk individuals, whereas the CHEST suggests against bridging unless in highly selected patients [30]. The 2022 ESC guidelines suggest bridging with eptifibatide or tirofiban may be applicable in rare cases when DAPT cannot be stopped, such as those with a very high risk of stent thrombosis, recurrent MI, or recent PCI; they also consider using a reversible P2Y_12_ inhibitor, ticagrelor, in which platelet function recovers faster after interruption than with other P2Y_12_ inhibitors and ASA [29]. In comparison, the Japanese and Australasian guidelines do not recommend bridging at all [66,67]. In the absence of consensus guidelines or a robust body of evidence, we suggest bridging may be considered in those at especially high thrombotic risk, with cangrelor as the preferred agent, if available.

**Figure 2:**
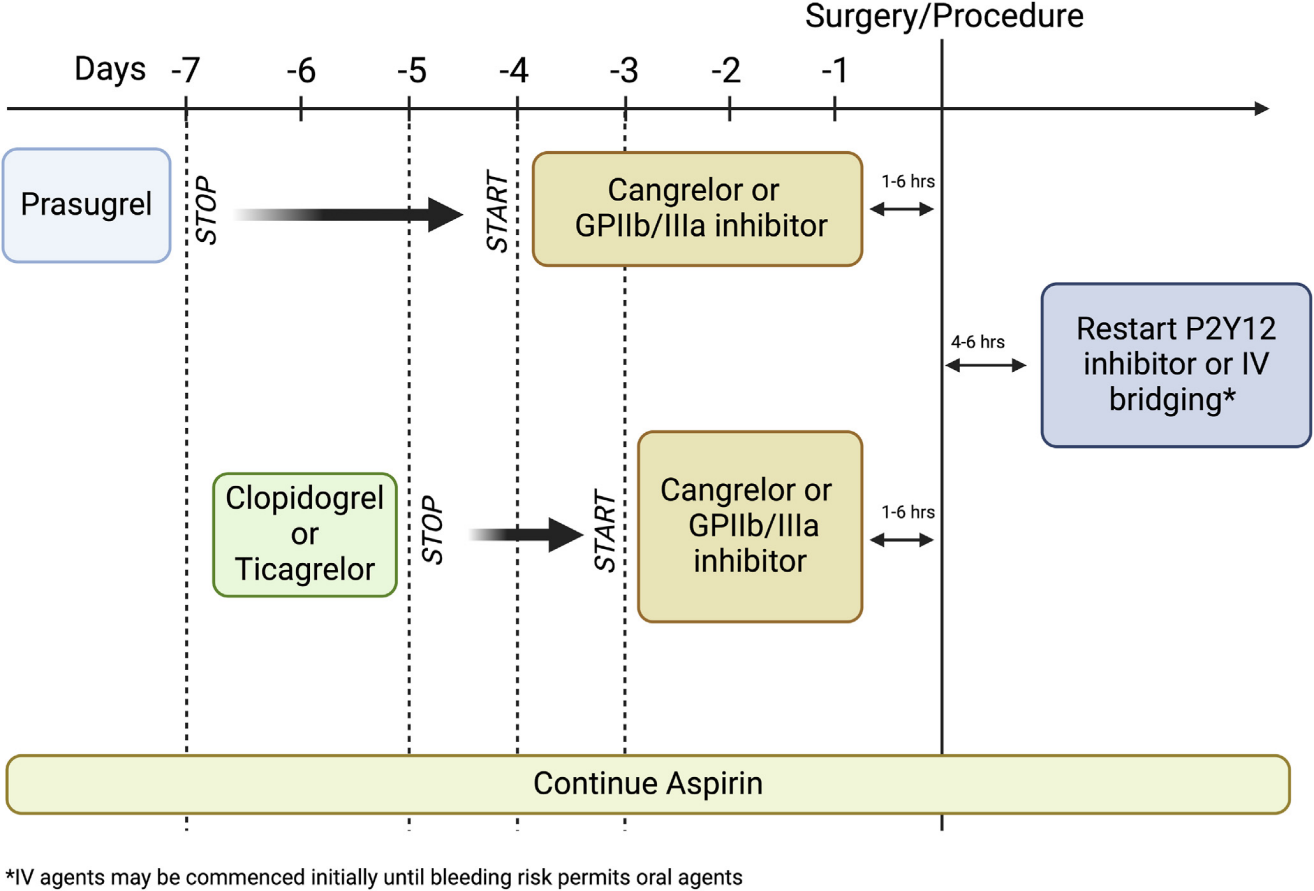
Bridging therapy for patients on antiplatelet agents at high risk of thrombosis. GP, glycoprotein; IV, intravenous.

An approach to bridging is shown in Figure 2.

## 6 Emergency Surgery

Unlike warfarin or direct oral anticoagulants, there are no reversal agents specific to antiplatelet drugs. When patients taking DAPT require emergency surgery after immediate cessation of the P2Y_12_ inhibitor, nonspecific prohemostatic management options include platelet transfusion or administering tranexamic acid and/or desmopressin (DDAVP). Whether this strategy is likely to improve or potentially restore hemostasis is dependent upon the P2Y_12_ inhibitor, timing of last antiplatelet dose, and comorbidities.

The evidence in support of platelet transfusion in patients taking P2Y_12_ inhibitors is sparse but suggests that platelet transfusion may be more beneficial in those taking clopidogrel than the other agents [68,69]. Most available data are from *in vitro* or *ex vivo* studies in which uninhibited platelet-rich plasma from healthy subjects was added to samples containing antiplatelet agents. For example, clopidogrel-induced platelet inhibition can be decreased with the addition of 40% noninhibited platelets from platelet-rich plasma [68]. In contrast, prasugrel-induced platelet dysfunction is only partially corrected by 60% noninhibited platelets, and ticagrelor-treated samples did not improve at all. These data, while useful, give no guidance regarding the optimum platelet dose for clinical use [69].

A study of patients receiving antiplatelet agents who presented with intracranial hemorrhage or the need for emergency neurosurgery reported that an average dose of 0.12 IU/kg of platelets effectively restored platelet function using the VerifyNow (Werfen USA LLC) P2Y_12_ test in patients receiving aspirin monotherapy but did not improve clopidogrel-induced dysfunction sufficiently to restore hemostasis [70]. Similarly, the APTITUDE-CABG study reviewed the impact of platelet transfusion on platelet reactivity in patients taking DAPT experiencing excessive surgical bleeding. An average of 5.5 ± 2.5 platelet concentrates (at 0.7 × 10^11^ platelets per concentrate) were transfused. Platelet reactivity index improved significantly following transfusion in those receiving clopidogrel but not for prasugrel- and ticagrelor-treated patients [71]. It seems likely that larger doses may be required for those treated with prasugrel than clopidogrel, but formal guidelines are lacking in this area. The issue is further compounded by the fact that the size of platelet concentrates varies from country to country. In the United Kingdom, for example, the minimum requirement for apheresis platelets is 2.4 × 10^11^, with an average of 3 × 10^11^ per unit [72]. The 5.5 units administered in the APTITUDE-CABG study thereby equate to 1 to 1.5 units.

In order for platelet transfusions to neutralize the effects of the P2Y_12_ inhibitors, the active metabolites of these drugs must be at sufficiently low levels in circulating plasma at the time of transfusion. Clopidogrel and prasugrel are irreversible P2Y_12_ inhibitors; however, their active compounds are only detectable for around 30 minutes and 4 hours after ingestion, respectively [39]. *In vitro* studies have reported minimal functional platelet recovery if donor platelets are added to blood samples obtained 2 hours after prasugrel administration. The authors, therefore, suggested that 6 hours should have elapsed prior to transfusion in these patients [73]. The same approach has been adopted by the French Working Group on Perioperative Haemostasis guidelines, which also suggest waiting 6 hours in clopidogrel-treated patients where possible [39].

However, while ticagrelor is a reversible P2Y_12_ inhibitor, unlike clopidogrel and prasugrel, it is directly active without the need for metabolic activation. Moreover, both ticagrelor and its first active metabolite remain in the circulation for up to 24 hours [[73], [74], [75]]. In the preclinical setting, studies have shown promise only if platelets are administered 24 hours or more after ticagrelor cessation [74,76]. A case report of a patient requiring emergency reversal of DAPT following intracranial hemorrhage demonstrated effective reversal of aspirin effect but failure to improve the platelet reactivity index (vasodilator stimulated phosphoprotein) 28 hours after ticagrelor ingestion, despite transfusion of 8.5 × 10^11^ platelets (approximately 3 units) [77].

**Figure 3:**
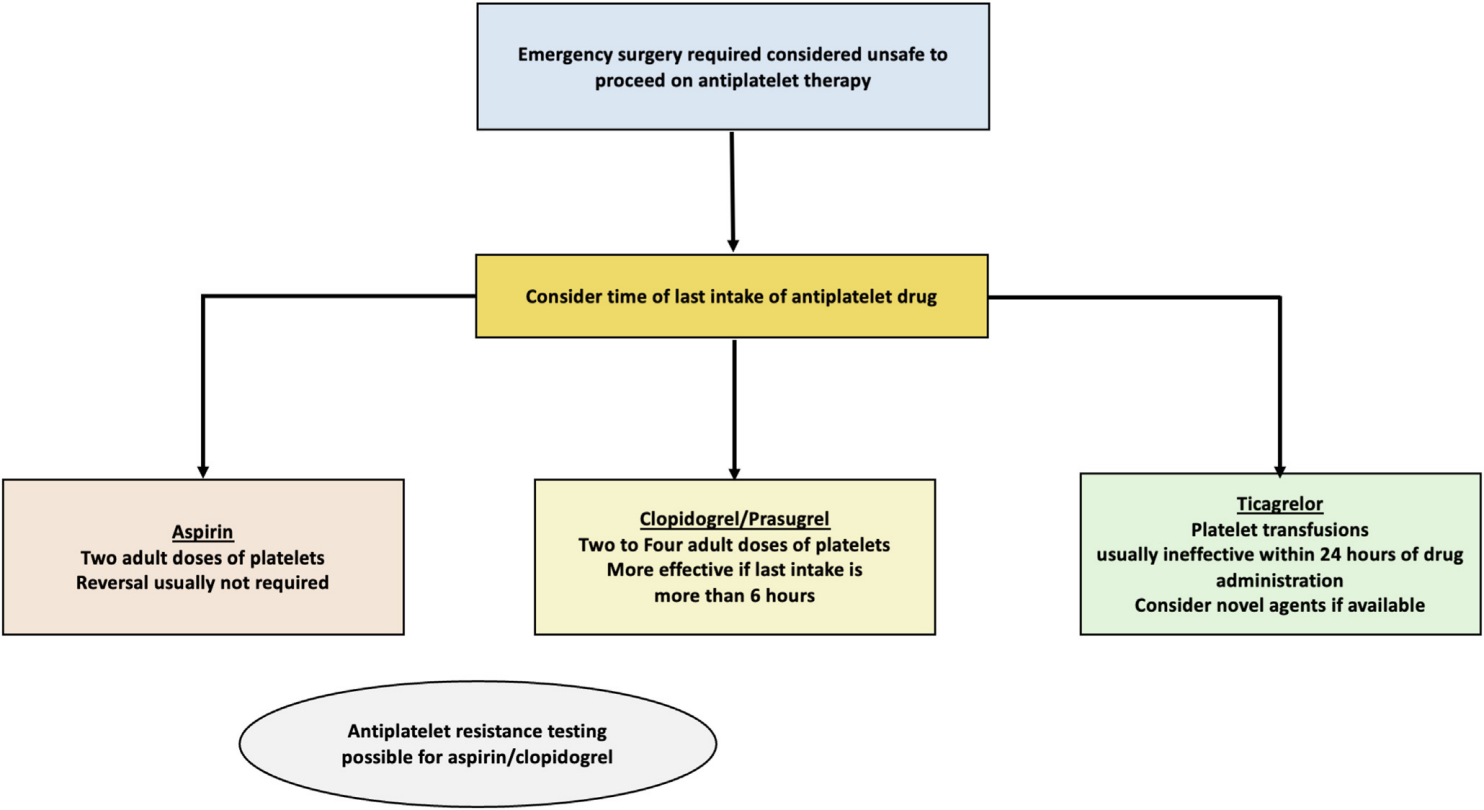
Algorithm for managing urgent surgery while receiving antiplatelet agents.

In the event of life- or limb-threatening bleeding in those taking DAPT or the need for emergency surgery, aspirin can be effectively reversed by platelet transfusion. Doing so for the P2Y_12_ inhibitors requires a higher dose of platelets, more so for prasugrel than clopidogrel, and to ideally wait for 6 hours following the last dose. The optimal dose is not known; however, 2 to 4 units would seem reasonable based upon the available published evidence. Ticagrelor cannot be effectively reversed by platelet transfusion while active metabolite remains in the circulation (see Figure 3).

### 6.1 Other approaches for ticagrelor reversal

Given the inability of platelet transfusion to neutralize the effect of ticagrelor, alternative approaches are clearly required. DDAVP has not been shown to be effective in this setting, although its use remains in guidelines for intracranial hemorrhage, and the recent phase 2 feasibility study, DASH, demonstrates the ongoing interest in use of DDAVP in this area [[78], [79], [80]]. Equally, recombinant-activated factor VII reduced bleeding in a murine model [81]; however, its use is associated with an increase in arterial thromboembolic events and should generally only be considered as a final resort [82]. Tranexamic acid has been shown to ameliorate inhibition of ADP-induced platelet aggregation using the Multiplate device in patients receiving clopidogrel [83]; however, it had no impact on corresponding thromboelastography-based parameters in ticagrelor-treated samples [84].

Other potential strategies include platelet-mimicking nanosponges, which competitively bind to antiplatelet agents. These nanosponges have a platelet membrane shell and inert perfluorocarbon inner core. They can therefore bind to antiplatelet drugs without the corresponding antiplatelet effect. They have shown promising results in murine models at reversing both ticagrelor and tirofiban-induced antiplatelet effect [85]. Use of external devices to remove antiplatelets from circulation has also been postulated. The CytoSorb (Cytosorbents Corporation) system contains absorbent polymer beads that can remove hydrophobic substances, such as ticagrelor, during cardiopulmonary bypass surgery [86]. Two studies of CytoSorb in ticagrelor-treated patients requiring emergency cardiac surgery, namely ticagrelor CytoSorb haemoadsorption (NCT04131959) and CyTation (NCT04625764), were terminated due to patient recruitment challenges, in part resulting from the SARS-CoV-2 pandemic.

## 7 Role of Point-of-Care Tests

Point-of-care (POC) testing to guide transfusion during cardiac surgery is now recommended in some consensus guidelines [87], with studies such as the Transfusion Avoidance in Cardiac Surgery trial in over 7000 patients demonstrating the potential value of viscoelastic testing to reduce transfusion burden and major hemorrhage [88]. This may be especially relevant for patients taking clopidogrel and prasugrel, which are prodrugs that are converted into their active metabolites by the cytochrome P450 CYP2C19 enzyme and because single nucleotide polymorphisms within the *CYP2C19* gene can significantly affect drug metabolism, leading to variable levels of observed platelet inhibition [89]. Platelet function testing can identify patients with high levels of ADP reactivity despite DAPT who are at increased risk of thrombotic events [90,91]. They may also be of use in patients requiring surgery by predicting bleeding risk and identifying patients who can safely proceed to surgery earlier after drug cessation. There are a number of potential tests described elsewhere [9,92], which are user-dependent and not directly comparable.

The randomized prospective TARGET-CABG study used thromboelastography-platelet mapping to determine level of ADP inhibition in 180 patients who required CABG and were either taking DAPT with clopidogrel or ASA monotherapy. Surgery was scheduled according to the maximum amplitude, with a delay of 24 hours, 3 to 5 days, and >5 days for maximum amplitude results of >50 mm, 35 to 50 mm, and <35 mm, respectively. The average time to surgery in the DAPT cohort was 2.3 days compared with the recommended 5, with no difference in bleeding or transfusion requirements between clopidogrel-treated and clopidogrel-naive cohorts [93]. Another study used platelet function testing (PFA-100, Siemens Healthineers) in 100 clopidogrel-treated patients prior to CABG and compared their outcomes with 100 patients undergoing CABG 5 days after clopidogrel cessation (standard of care) and 100 patients who had not received clopidogrel. The choice of POC was the PFA-100 system using a P2Y cartridge. Assessment of platelet function was associated with a significant reduction in postoperative bleeding and lower packed red cell requirements compared with the standard of care arm and no significant difference compared with the clopidogrel-naive group. Patients waited on average 3.6 days for surgery [94]. Case reports suggest this approach may also be useful for prasugrel-based DAPT [95].

One group assessed preoperative POC (Multiplate) in 226 patients undergoing CABG, of whom 140 were receiving clopidogrel-based DAPT and 86 ticagrelor-based therapy. Bleeding (defined as chest tube loss of more than 450 mL at 6 hours after surgery) was significantly less common among patients with recovered platelet function before surgery irrespective of antiplatelet regimen, with bleeding predicted by ADP test results of <46 U (*P* = .001) [96]. Reduced ADP- and thrombin receptor activating peptide-induced platelet aggregation preoperatively also predicted severe bleeding in 74 ticagrelor-treated surgical patients with a suggested cutoff level of 25 U and 100 U for ADP and thrombin receptor activating peptide, respectively. This group also reported that postoperative POC testing could similarly identify patients at increased risk of hemorrhage [97]. Although these and other similar studies have identified potential thresholds for bleeding risk, differences in choice of POC modality as well as antiplatelet regimen limit generalization of results. However, if available, these tests may be of use. This may be particularly applicable to patients who require emergency surgery, in whom being able to safely proceed to theater before the routinely suggested drug-free window period has elapsed holds clear advantages.

An approach to emergency surgery is shown in Figure 3.

## 8 Conclusion

Despite the recent reduction in use of ASA as monotherapy for the management of patients with CVD [98], DAPT use continues to rise [7]. This added complexity in antiplatelet therapy has implications on perioperative management, warranting consideration of temporary interruption of 1 or both antiplatelet drugs. Practice guidelines, in general, provide weak and, sometimes, inconsistent recommendations regarding elective procedures in stable patients, reflecting the limited evidence base. The uncertainty in best practices is greater among those requiring emergency surgery. Unresolved questions include the need for and quantity of platelet transfusion and how to restore platelet function in a timely manner, depending on whether patients are receiving a partially reversible (ticagrelor) or irreversible (clopidogrel, prasugrel) antiplatelet agent. The role of bridging in selected high-risk patients is yet to be defined by designed clinical trials, and uncertainty remains as to which patients, if any, benefit from antiplatelet bridging and which agent and dosing to use. POC testing to assess platelet function, though promising, needs further study, especially outside of a CABG setting. While updated results from the REVERSE-IT study are highly anticipated, there are otherwise very few ongoing clinical trials specifically aimed at addressing these questions, which may continue to plague clinicians for years to come.
